# Risk factors and incidence of surgical wound infection after stoma reversal: A systematic review and meta-analysis

**DOI:** 10.1371/journal.pone.0328344

**Published:** 2025-07-16

**Authors:** Siruo Li, Ziyi Zhou, Feixia Wang, Weizhen Li, Chaoxu Liu, Lu Li

**Affiliations:** 1 Zhejiang University, Hangzhou City, Zhejiang Province, China; 2 Department of Nursing, The First Affiliated Hospital of Zhejiang University, Hangzhou City, Zhejiang Province, China; 3 Department of Colorectal Surgery, The First Affiliated Hospital of Zhejiang University, Hangzhou City, Zhejiang Province, China; Shuguang Hospital, CHINA

## Abstract

To determine the incidence and risk factors of incisional infection following stoma reversal surgery. As of July 30, 2024, an extensive literature search was conducted on databases including PubMed, Web of Science, Cochrane, Embase, and OpenGrey. The certainty of evidence was evaluated using the Grading of Recommendations, Assessment, Development, and Evaluations (GRADE) approach. Data analysis was performed using Stata 14.0.20 articles were included, with a total sample size of 8542, including 723 patients with incision infection. The incidence of incision infection was 12%(95%CI:0.094–0.145). The results identified 5 patient-related risk factors of incisional infection, including stoma type (OR: 3.06, P = 0.015), inflammatory bowel disease (OR: 1.91, P = 0.012), Body Mass Index (BMI; OR: 1.12, P < 0.01), period from stoma creation (OR: 0.18, P = 0.012), and surgical site infection (SSI) after primary surgery (OR: 3.57, P < 0.01), and 3 surgery-related risk factors, including subcutaneous drainage (OR: 0.26, P = 0.019), suture method (OR: 4.83, P < 0.01), operation time > 60 min (OR: 4.33, P < 0.01), operation time (continuous variable, OR: 1.004, P < 0.01). Clinical staff can refer to the influential factors in this study to reduce the incidence of incision infection.

## Introduction

Ostomy is one of the common treatment modalities for colorectal cancer, diverticular disease, and inflammatory bowel disease, especially colorectal cancer. The temporary stoma plays a role in diverting feces and reducing anastomotic contamination. It can reduce the severity of postoperative complications and mortality rate [1]. 3 to 6 months following ostomy, patients undergo stoma reversal to return the bowel and restore physiologic excretory access. With the rising incidence of colorectal cancer, the number of patients undergoing temporary stoma and stoma reversal is also increasing [2]. The complication rate of stoma reversal is high, ranging from 6.7% to 40%, of which SSI is the most common complication [3–5].

The reported incidence of SSI after stoma reversal varies widely. The incidence of wound infection after stoma reversal ranges from 2–41% due to peristomal skin complications resulting in compromised skin integrity and colonization by pathogenic microorganisms [6,7]. SSI will not only delay the speed of incision healing, but also further develop into incision dehiscence, incisional hernia, and even septic shock. At the same time, it will also prolong the hospitalization time of patients, increase the readmission rate and the rate of secondary surgery, and increase the social medical burden [1,8]. Early detection of high-risk patients and prompt intervention are key to lowering the incidence of SSI [9].

It has been shown that BMI affects the incidence of SSI after stoma reversal [10]. However, another study found that BMI was not an influencing factor [3]. Given the conflicting findings and the small sample size of individual studies, a meta-analysis is necessary to assess the results of the current study fully. Therefore, this study aims to investigate the incidence of SSI after stoma reversal and to identify the risk factors influencing SSI after stoma reversal.

## Methods

### Literature search

The search strategy and process of this study were reported according to PRISMA 2020 [11], the reporting specification guide for systematic evaluation and Meta-analysis. Our research was preregistered with PROSPERO(ID: CRD42023449934).

From the inception of the database to July 30, 2024, 2 researchers independently and comprehensively searched PubMed, Web of Science, Cochrane, and Embase for English-language studies. Additionally, the English language literature of OpenGrey, a gray literature database, was searched from the beginning of the database to January 6, 2025. The search terms were as follows:(‘ileostomy’, ‘colostomy’, ‘ostomy’, ‘loop ileostomy’, ‘diverting ileostomy’, ‘surgical stomas’, ‘stoma’); (‘closure’, ‘reversal’); (‘complications’, ‘morbidity’); (‘surgical wound infection’, ‘Surgical Wound Infection’, ‘Surgical Site Infection’, ‘Postoperative Wound Infection’, ‘SSI’); (‘Risk Factor*’, ‘Influencing Factor*’, ‘predictor’). The search strategy for all databases was presented in S 1 File. In addition, references to included studies were manually searched for other relevant articles that were not captured by the initial search.

### Inclusion and exclusion criteria

Inclusion criteria: ① Study type: cross-sectional study, retrospective or prospective cohort study, case-control study; ② Patient characteristics: patients ≥18 years of age who have undergone stoma reversal surgery;③ The study reported the influencing factors of incision infection after stoma reversal and the corresponding OR or RR values and 95% CI values, ④ Literature type: Conference papers, academic papers.

Exclusion criteria: ① Articles for which full text is unavailable;② Reviews, study protocols, letters, animal experiments, and case reports;③ Studies with insufficient data.

### Diagnostic criteria

Diagnostic criteria for surgical incision infections refer to the Centers for Disease Control and Prevention [12]. Surgical incision infections include superficial incision infections, deep incision infections, and organ infections. Superficial incision infections occur in the skin and subcutaneous tissue within 30 days of surgery. Deep incision infection is a surgical-related infection involving deep soft tissue (deep fascia and muscle) of the incision within 30 days after implant-free surgery and within 1 year after implant surgery (such as artificial heart valves, artificial blood vessels, mechanical hearts, and artificial joints).

### Ethical statement

This study is a systematic review and meta-analysis, and ethical statements are not suitable.

### Data extraction

2 independent reviewers (Siruo Li; Ziyi Zhou) screened the abstracts of all identified studies using Endnote. Studies that were likely to be included in the review would be obtained in full-text form and assessed by the same reviewers (Siruo Li; Ziyi Zhou). Any disagreements at any stage of study selection would be resolved by consensus or referral to a third reviewer (Weizhen Li). Reasons for rejection at the full-text screening stage will be presented in S1 File. Data for inclusion in the literature of this study were extracted by two researchers (Siruo Li; Ziyi Zhou) and organized using Excel. The extracted data included: first author, publication year, country, study year, sex, sample size, infection rate, follow-up time, study design, risk factors, and Newcastle-Ottawa scale (NOS) score.

### Quality assessment

The quality of the screened articles was evaluated by two independent researchers. The Newcastle-Ottawa Quality Assessment Scale (NOS) was used to rate the quality of literature included in the cohort and case-control studies, with a score of 1–3 as low-quality literature, 4–6 as moderate-quality literature, and 7–9 as higher-quality literature [13]. The American Agency for Healthcare Quality and Research (AHRQ) Criteria for Evaluating the Quality of Cross-Sectional Studies was used to score cross-sectional studies, with a score of 0–3 as low-quality literature, a score of 4–7 as moderate-quality literature, and a score of 8–11 as higher-quality literature [14]. The Grading of Recommendations, Assessment, Development, and Evaluation (GRADE) has been used to rate the overall certainty or quality of evidence for each of the pooled risk factors, and categorized them as either “high,” “moderate,” “low,” or “very low” (S1 File) [15]. Consensus was reached for final inclusion of the studies, and if there was disagreement the decision to include was left to the judgment of a third party.

### Statistical analysis

Stata14.0 was used to conduct the meta-analysis. Using the 95% credible interval (95% CI) and odds ratio (OR), the influencing factors were pooled to create effect sizes. The degree of heterogeneity was evaluated using the I^2^ test; if P > 0.1, I^2^ < 50%, there was no heterogeneity, and a fixed-effects model was employed; if P < 0.1, I^2^ > 50%, there was significant heterogeneity, and a random-effects model was used, along with sensitivity analysis or subgroup analysis. For SSI after stoma reversal incidence subgroup analysis of stoma type was performed. Publication bias analysis was performed when ≥10 papers were included.

## Results

### Study selection

**Fig 1:**
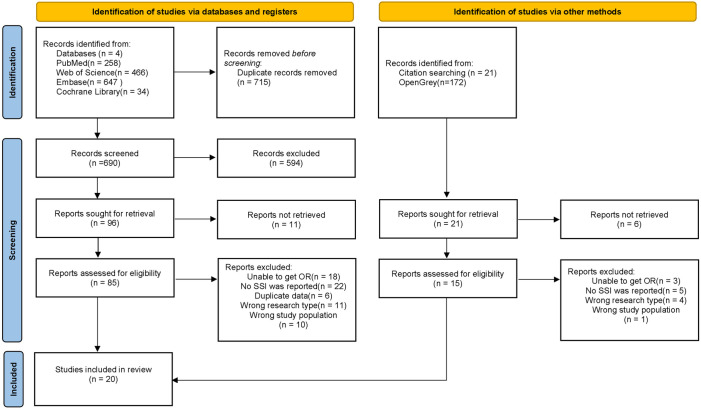
The procedure of literature selection.

The initial search of the database yielded 1598 documents, and 715 documents were obtained after eliminating duplicate publications. Following reading the titles and abstracts, 100 documents were obtained and assessed at the full text, and 20 of these were included after full-text reading (Fig 1).

### Study characteristics

Characteristics of the 20 retained studies are presented in Table 1. A total sample size of 8542 cases were enrolled in the studies and 723 patients with incisional infections were finally included. Among the studies included, the most common country of origin was the USA(n = 7), the remaining studies were conducted in Australia (n = 3), Germany (n = 1), Mexico (n = 1), and 8 studies from Asia. 32 potential risk factors were extracted for analysis.

**Table 1:** The characteristics of the 20 included studies.

First Author	Publication Year	Country/ Study year	Gender (male/ female)	Sample size	Infection rate	Study type	Follow-up time	Risk factors	Newcastle-Ottawa scale (NOS)			
									Selection	Comparability	Exposure/ Outcome	Quality scores
Takashi [16]	2010	Japan 2005-2009	78/47	125	20(16%)	Retrospective cohort study	30 days	Gender, Surgical site infection after primary surgery	***	*	**	6
Yong Joon [17]	2014	Korea 2010-2011	99/58	157	9(5.73%)	Retrospective cohort study	30 days	Smoke, BMI, Suture method, Skin anastomosis	***	**	**	7
Naseem [10]	2012	Australia 2003-2009/2010-2011	90/52	142	15(10.7%)	Retrospective cohort study	30 days	Suture method, Stoma type, BMI, Antibiotic prophylaxis	****	**	*	7
Shin [18]	2010	Japan 2008	38/13	51	12(23.5%)	Prospective cohort study	30 days	Stoma type, Age, Skin anastomosis	***	**	***	8
Cribb [19]	2021	Australia 2012-2020	81/45	126	5(4%)	Retrospective cohort study	30 days	Period from stoma creation, Gender, Chemoradiotherapy, Radio chemotherapy	****		**	6
Yamamoto [6]	2018	Japan 2000-2014	84/44	128	21(16.4%)	Prospective cohort study	30days	Smoke, BMI, Diabetes, Suture method, The modified Glasgow Prognostic Score	***	*	**	6
Bhama [20]	2017	America 2010-2015	888/849	1737	183(10.5%)	Retrospective case-control study	30 days	Coagulation disorders, Operation time, Smoke	***	*	**	6
Zhu [21]	2022	China 2015-2020	130/71	201	37(18.4)	Retrospective case-control study	30 days	Nutrition risk screening, C-reaction protein, Suture method	***	*	*	5
Li [22]	2014	America 2005-2011	134/12	146	32(22%)	Retrospective cohort study	30 days	BMI, Suture method	***	*	***	7
Paula [3]	2020	America 2015-2016	1059/826	1885	116(6.2%)	Retrospective cohort study	30 days	Gender, Age, BMI, Operation time, Cancer, Inflammatory bowel disease, Diverticulosis, Dyspnea	***	*	*	5
Liang [23]	2013	America 2005-2011	121/7	128	46(36%)	Retrospective Case-control study	30 days	History of fascia cracking, Stoma type, Subcutaneous fat thickness, Race	***	*	*	5
Kamada [24]	2021	Japan 2013-2020	111/71	182	53(29%)	Retrospective cohort study	30 days	Gender, Smoke, Subcutaneous fat thickness, Surgical site infection after primary surgery, Period from stoma creation, Suture method, Amount of blood loss during surgery, Subcutaneous drainage	***	*	***	7
Vergara-Fernández [25]	2019	Mexico 2010-2018	67/69	136	9(7%)	Retrospective cohort study	30 days	Incision type	***	**	*	6
Chu [26]	2015	America 2006-2011	284/244	528	36(6.8%)	Retrospective Case-control study	30 days	Physical status (ASA class), Smoke, Stoma type, Incision type, Inflammatory bowel disease, Cancer, History of fascia cracking, Subcutaneous drainage	***	**	*	6
Pan [27]	2015	China 2006-2013	145/100	245	21(8.6%)	Retrospective cohort study	30 days	Subcutaneous drainage, Operation time, Postoperative complications	****	**	**	8
Nyandoro [28]	2022	Australia 2015-2021	34/72	106	20(18.9%)	Retrospective cohort study	30 days	Surgical-site antisepsis, Antibiotic prophylaxis, Operation time, Period from stoma creation	****	**	*	7
Savigny [29]	2023	Germany 2009-2019	115/185	300	19(6.3%)	Retrospective cohort study	30 days	BMI, Days until first bowel movement	***	**		5
Mirande [30]	2023	America 2012-2021	324/324	648	37(5.7%)	Retrospective Case-control study	30 days	Inflammatory bowel disease, Diverticulosis, Operation time	***	**	*	6
Wong [31]	2005	America 1983-2002	889/615	1504	23(1.5%)	Retrospective cohort study	30 days	Period from stoma creation	***	**	**	7
Ohara [32]	2024	Japan 2019-2022	45/22	67	9(13%)	Retrospective cohort study	30 days	Stoma type	**	*	**	5

Abbreviation: BMI, body mass index; ASA, American Society of Anesthesiologists.

### Quality assessment

The quality of the literature included was generally high, with 8 studies being of high quality and 12 of moderate quality (Table 1). The NOS average score was 6.3.

### The overall incidence of SR-SSI(Stoma Reversal-Surgical Site Infection)

**Fig 2:**
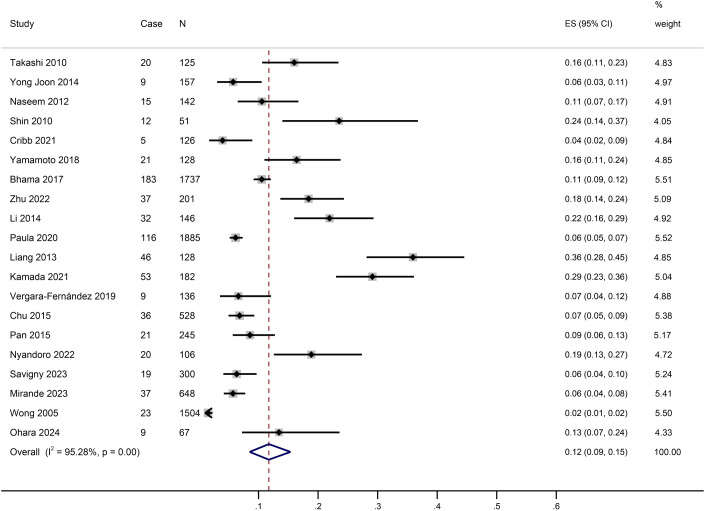
The overall incidence of incisional infections after stoma reversal surgery.

Meta-analysis was performed on the 20 included studies. The results showed that the incidence of SR-SSI was 12%(95% CI:9%−15%, I^2^ = 95.28%), as shown in Fig 2.

### Risk factors

32 risk factors were extracted from the 20 included studies, of which 15 were meta-analyzed. The remaining 17 could not be meta-analyzed. Of these 17 risk factors, age, antibiotics prophylactic, and skin anastomoses were not quantitatively analyzed due to inconsistent definitions that would have generated errors. The remaining 14 risk factors were all reported in only 1 study and could not be quantitatively analyzed. The results of the meta-analysis can be seen in Table 2.

**Table 2:** Summary of risk factors for surgical site infection after stoma reversal surgery.

Potential risk factors	No. of study	Pooled OR	LL 95%CI	UL 95%CI	P value	I^2^(%)	FE/RE
Subcutaneous drainage	3	0.264	0.087	0.804	0.019	50.4	RE
Suture method*	6	4.83	2.83	8.26	0	0	FE
Incision type	2	2.798	0.794	9.853	0.109	49.9	RE
History of fascia cracking	2	3.921	0.497	30.944	0.195	57.6	RE
Stoma type*	5	3.062	1.246	7.522	0.015	58.5	RE
Inflammatory bowel disease	3	1.911	1.151	3.173	0.012	0	FE
Cancer	2	1.081	0.599	1.950	0.796	0	FE
Operation time(> 60 min)	3	4.334	2.917	6.44	0	0	FE
Operation time(continued)	2	1.004	1.002	1.007	0	38.6	FE
Period from stoma creation*	2	0.181	0.048	0.684	0.012	0	FE
Smoke	5	1.546	0.671	3.561	0.306	68.7	RE
BMI(≥25 kg/m^2^)	3	1.779	0.82	3.859	0.145	70.6	RE
BMI(continued)	2	1.116	1.077	1.157	0	20.4	FE
Gender*	4	1.251	0.412	3.796	0.692	74.4	RE
Surgical site infection after primary surgery	2	3.573	1.633	7.817	0.001	0	FE

Abbreviation: OR: odds ratio; LL: lower limit; UL: upper limit; CI: confidence interval; BMI: body mass index; FE: fixed model; RE: random model

* Reported ORs were adjusted.

### Patient-related risk factors

#### Stoma type.

**Fig 3:**
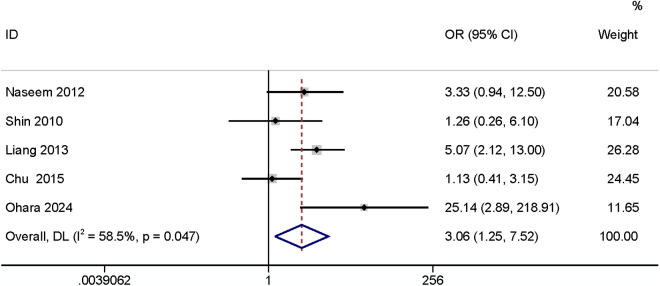
Stoma type.

Five articles [10,18,23,26,32] comparing ileostomies with colostomies found that the type of stoma can have an impact on SR-SSI (OR: 3.06, 95% CI: 1.25–7.52, P = 0.015, I2 = 58.5%, Fig 3; GRADE assessment: very low).

#### Inflammatory bowel diseas.

**Fig 4:**
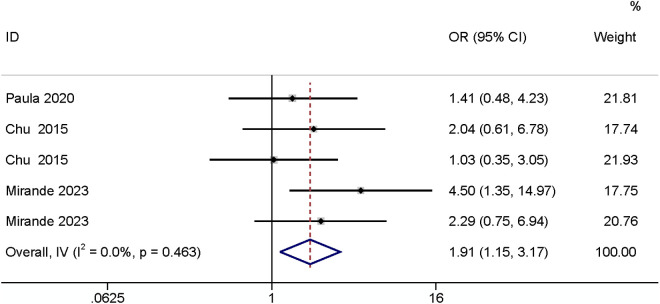
Inflammatory bowel disease.

A meta-analysis of three studies [3,26,30] showed that it would increase the risk of SSI when the underlying disease is inflammatory bowel disease (OR: 1.91, 95% CI: 1.15–3.17, P = 0.012, I^2^ = 0; Fig 4; GRADE assessment: low).

#### Period from stoma creation.

**Fig 5:**
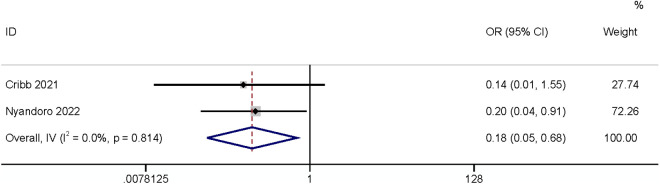
Period from stoma creation.

**Fig 6:**
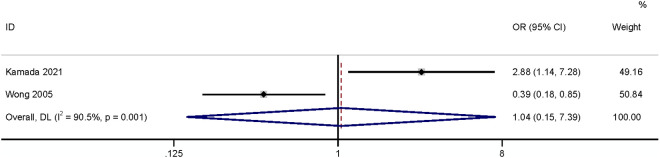
Period from stoma creation (continued).

Four studies reported periods from stoma creation, two of which defined [24,31] this factor as a categorical variable and the other two as a continuous variable. The results showed that the period from stoma creation (categorical variable) had a relationship with SSI (OR: 0.18,95% CI: 0.05–0.68, P = 0.012, I^2^ = 0; Fig 5; GRADE assessment: low). A meta-analysis of two studies [19,28] found that the period from stoma creation (continuous variable) had no relationship with SSI (OR: 1.04,95% CI: 0.15–7.39, P = 0.97, I^2^ = 97%, Fig 6; GRADE assessment: very low).

#### BMI.

**Fig 7:**
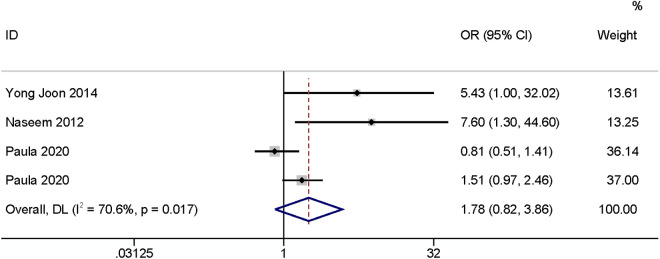
BMI  ≥ 25 kg/m^2^.

**Fig 8:**
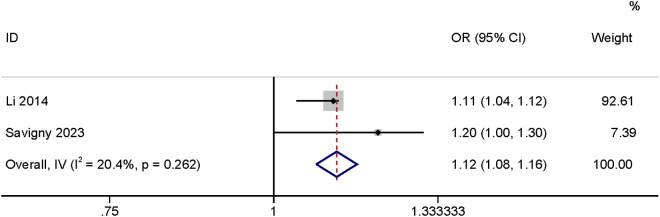
BMI-continued.

Five studies reported BMI, three of which [3,10,17] took BMI ≥ 25 kg/m^2^ (OR: 1.78, 95% CI: 0.82–3.86, P < 0.15, I^2^ = 70.6%; Fig 7; GRADE assessment: very low). It had no effect on the occurrence of SSI. The other two papers [22,29] defined BMI as a continuous variable, at which point the results indicated that BMI had an impact on SSI (OR: 1.12, 95% CI: 1.08–1.16, P < 0.01, I^2^ = 20.4%; Fig 8; GRADE assessment: low).

#### Surgical site infection after primary surgery.

**Fig 9:**
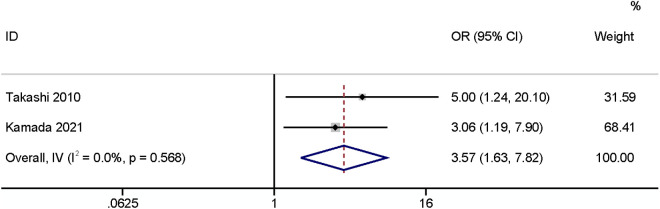
Surgical site infection after primary surgery.

A meta-analysis of two studies [16,24] found that surgical site infection after primary surgery had a significant relationship with SSI (OR: 3.57,95% CI: 1.63–7.82, P < 0.01, I^2^ = 0; Fig 9; GRADE assessment: low).

#### Subcutaneous fat thickness.

**Fig 10:**
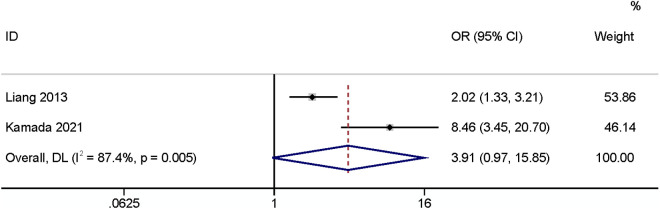
Subcutaneous fat thickness.

A meta-analysis of two studies [23,24] found that subcutaneous fat thickness had no relationship with SSI (OR: 3.91,95% CI: 0.97–15.85, P = 0.06, I^2^ = 87.4; Fig 10; GRADE assessment: very low).

#### History of fascia cracking.

**Fig 11:**
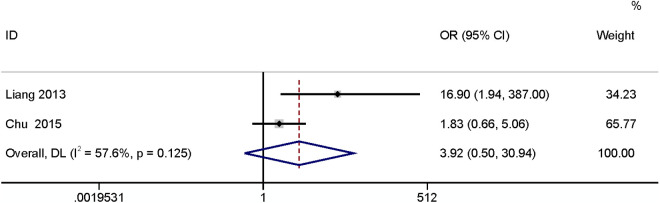
History of fascia cracking.

The meta-analysis of two studies [23,26] found that history of fascia cracking had no relationship with SSI (OR: 3.92,95% CI: 0.50–30.94, P = 0.20, I^2^ = 57.6; Fig 11; GRADE assessment: very low).

#### Cancer.

**Fig 12:**
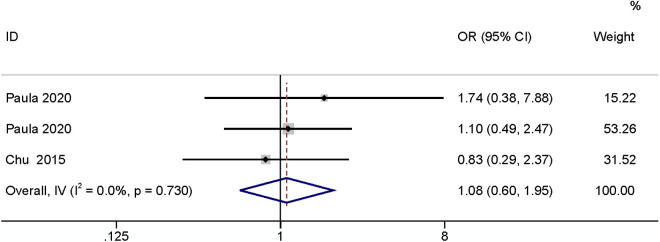
Cancer.

The meta-analysis of two studies [3,26] found that cancer had no relationship with SSI (OR: 1.08,95% CI: 0.60–1.95, P = 0.80, I^2^ = 0; Fig 12; GRADE assessment: low).

#### Diverticulosis.

**Fig 13:**
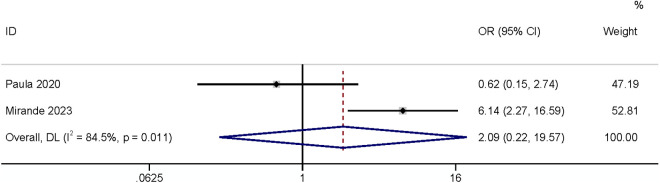
Diverticulosis.

The meta-analysis of two studies [3,30] found that diverticulosis had no relationship with SSI (OR: 2.09,95% CI: 0.22–19.57, P = 0.52, I^2^ = 84.5; Fig 13; GRADE assessment: very low).

#### Smoke.

**Fig 14:**
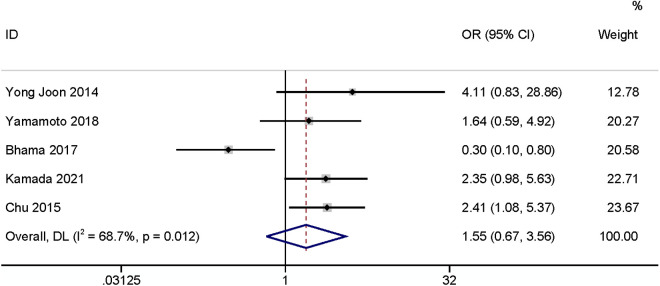
Smoke.

The meta-analysis of five studies [6,17,20,24,26] found that smoke had no relationship with SSI (OR: 1.55,95% CI: 0.67–3.56, P = 0.31, I^2^ = 68.7; Fig 14; GRADE assessment: very low).

#### Gender.

**Fig 15:**
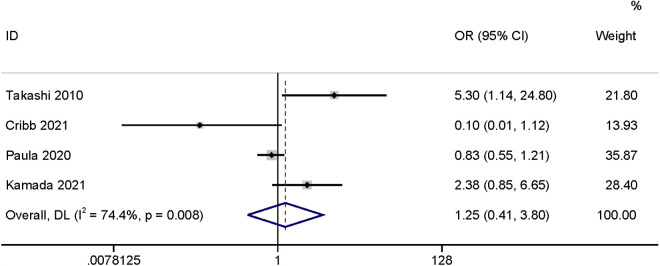
Gender.

The meta-analysis of four studies [3,16,19,24] found that gender had no relationship with SSI (OR: 1.25,95% CI: 0.41–3.80, P = 0.69, I^2^ = 74.4; Fig 15; GRADE assessment: very low).

### Surgery-related risk factors

#### Subcutaneous drainage.

**Fig 16:**
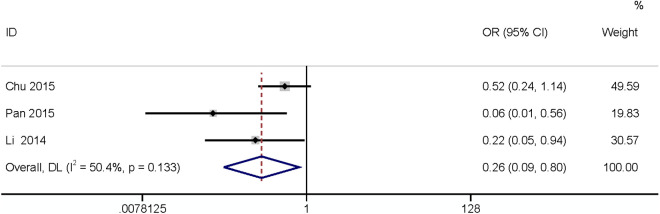
Subcutaneous drainage.

Three studies [24,26,27] reported subcutaneous drainage may affect SR-SSI. This study found that subcutaneous drainage was a protective factor. It can reduce the incidence of SR-SSI (OR: 0.26, 95% CI: 0.09–0.90, P = 0.019, I^2^ = 50.4%; Fig 16; GRADE assessment: low).

#### Suture method.

**Fig 17:**
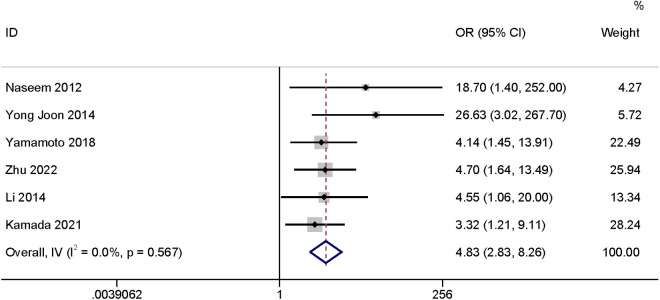
Suture method.

A total of six studies [6,10,17,21,22,24] revealed that the suturing method may have an impact on the occurrence of SSI. The results found that purse closure was related to SR-SSI (OR: 4.83, 95% CI: 2.83–8.26, P < 0.01, I^2^ = 0%; Fig 17; GRADE assessment: low).

#### Operation time.

**Fig 18:**
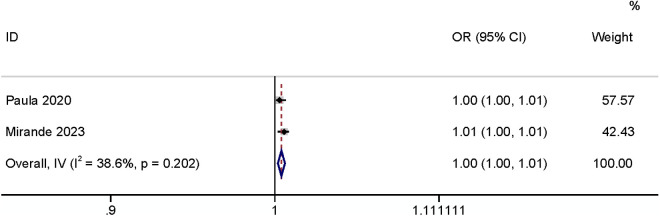
Operation time-continued.

**Fig 19:**
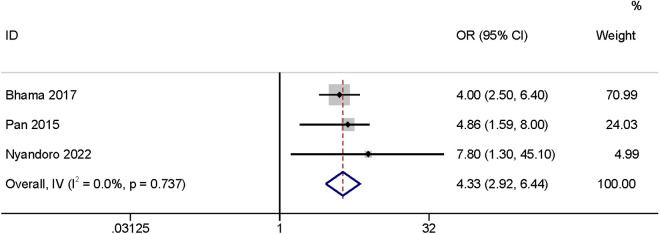
Operation time>60 minutes.

Five studies found that operation time may influence SSI incidence. Increased surgical duration elevated the risk of incidence of SR-SSI. Two papers [3,30] defined the operation time as a continuous variable and showed that it had a relationship with the occurrence of SSI (OR: 1.004, 95% CI: 1.002–1.007, P < 0.01, I^2^ = 38.6%; Fig 18; GRADE assessment: very low). Three papers [20,27,28] defined the operation time as a categorical variable, and the results showed that when the operation time was longer than 60 minutes it increased the risk of SSI (OR: 4.33, 95% CI: 2.92–6.44, P < 0.01, I^2^ = 0%; Fig 19; GRADE assessment: low).

#### Incision type.

**Fig 20:**
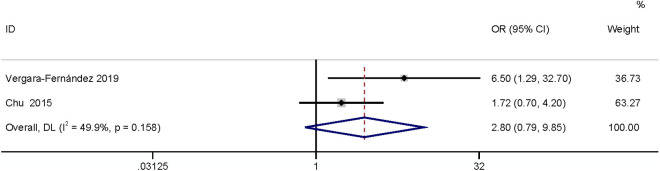
Incision type.

The meta-analysis of two studies [25,26] found that incision type had no relationship with SSI (OR: 2.80, 95% CI: 0.80–9.85, P = 0.11, I^2^ = 49.9; Fig 20; GRADE assessment: very low).

### Sensitivity analysis results

Sensitivity analyses of the incidence of SR-SSI were robust (S1 File). There was significant heterogeneity in both subcutaneous drainage (I^2^ = 50.4%) and stoma type (I^2^ = 58.5%), and sensitivity analysis for the items showed stable results for subcutaneous drainage and stoma type (S1 File). The heterogeneity of subcutaneous drainage may be related to the different methods of drainage, with 1 study investigated with a silicone duct connected to a squeezable, vacuum reservoir [27], and 1 used gauze [22], the other didn’t mention the specific way of drainage due to its study design [26]. The heterogeneity of stoma types may be due to the fact that one study adopted a closed suction drain and subcutaneous large-bite buried suture technique to reduce the incidence of SSI [32]. We performed sensitivity analyses for subcutaneous fat thickness, diverticulosis, and gender, and the results were robust (S1 File).

### Subgroup analysis and publication bias

A subgroup analysis of SR-SSI incidence was performed to investigate the effect of stoma type on SR-SSI incidence (S1 File). The results showed that the incidence of SR-SSI was higher in patients with colostomy. Publication bias analysis was performed on the incidence of SR-SSI, and the results can be seen in S1 File Egger publication bias test P = 0.008, which indicates some publication bias.

## Discussion

SSI is a common complication that can occur after stoma reversal, leading to negative outcomes such as prolonged hospitalization and surgical failure. While numerous influencing factors have been reported in the literature, the findings are often complex and inconsistent. Crucially, a comprehensive and systematic synthesis of these risk factors specifically for SR-SSI is lacking, and the true impact of many proposed factors remains unclear. This knowledge gap has hindered progress in both understanding the etiology and developing effective prevention strategies for SR-SSI. To address this critical need, our study undertakes a rigorous and comprehensive examination of the contributing factors for SR-SSI. The primary contribution of this work lies in its systematic integration and critical evaluation of the existing, often conflicting, evidence, providing a clearer and more consolidated picture of the key risk factors associated with SR-SSI. This foundational synthesis clarifies the current landscape and establishes a solid basis for future research and targeted clinical interventions.

### The overall incidence of SR-SSI

The incidence of SSI in this study was 12%, which aligns with other literature reports ranging from 0% to 33% [33–35]. This incidence with high heterogeneity is in the lower middle range. This may be related to differences in study design, diagnostic guidelines between regions, and level of care. The incidence of SSI in colorectal surgery in the United States is 1–3% [8], and the incidence of SSI in colorectal surgery in China is 5.6% [36]. The rate of SSI reported in this study is much higher than in other abdominal surgeries. In addition, being a second-stage procedure in a short period of time, susceptible to infection, as well as taking into account enteric nutrition, stoma reversal has special characteristics. This calls for increased attention to SSI after stoma reversal. We believe that our study will not only increase the attention of researchers to the complications of SSI after stoma reversal but also help clinicians to identify high-risk patients and take early interventions to prevent postoperative SSI, thereby reducing healthcare costs and improving patient outcomes.

### Risk factors

Postoperative drainage has been found to reduce the risk of infection, as demonstrated by the pooled results of the study. This is consistent with the results of Zhu et al. [37]. Commonly used clinical drainage materials include sterile silicone hose or sterile gauze. These materials enable the one-way flow of fluid through gravity or osmotic pressure, effectively flushing the area and reducing local pressure. However, there is no comparison of the effect of the two materials on the postoperative healing time of the incision. It is more of an intraoperative judgment of the amount of local fluid, and there is no uniform judgment standard. As for the suture method, linear sutures are associated with a nearly 5-fold increased risk of SSI compared with purse-string sutures. The purse-string suture with a hole in the middle can facilitate the timely drainage of the wounds. McCartan et al. [38] thus suggested that this method results in the presence of open wounds for a prolonged time, which may negatively affect the quality of life of the patient. However, Rondelli et al. [39] found that the length of patient hospitalization was not affected by the suture approach and that the total length of hospitalization could be lower than one-stage sutures due to the lower incidence of SSIs with purse-string sutures. This suggests that clinical practitioners should take a holistic approach when choosing sutures to benefit their patients.

Colostomy is considered a risk factor for SSI. The higher chance of incision infection after colostomy reversal compared to ileostomy reversal is consistent with the results of an earlier meta-analysis by Tilney et al. [40]. This may be related to the high bacterial load at the colonic site. In addition, colostomy reversal is more difficult than ileostomy reversal. The colon is characterized by a thinner intestinal wall and poorer blood supply, making the procedure relatively more difficult and longer, and the longer the procedure, the higher the chance of infection. Therefore, it is important to intervene in patients undergoing colostomy rejuvenation and to monitor the patient’s incision healing for early detection of signs of infection.

A study shows patients with inflammatory bowel illness have a greater incidence of SSI [41]. This aligns with the findings of the current investigation. As demonstrated by Mansfield et al. [42], the incidence of SSI in patients with inflammatory bowel disease is influenced by nutritional state and the use of hormone medicines. Hormonal therapy is a common treatment for people with inflammatory bowel disease, and it suppresses the patients’ immune systems. In addition, the patient may have pre-existing peritonitis and have low nutritional status as a result of the condition, both of which hinder the healing of the incision after surgery. Therefore, we need to pay special attention to patients with inflammatory bowel disease who undergo stoma reversal and take measures to prevent SSI.

Stoma reversal is typically performed 3–6 months after stoma surgery, but there is no clinical consensus on this interval. We found that incisional infection after stoma reversal decreased with increasing reversal interval. Early stoma reversal is safe, feasible, and reduces stoma-related complications, according to O’Sullivan et al. [43]. However, it increases postoperative complications, particularly incisional complications, possibly due to the patient’s abdominal cavity remaining adherent after one-stage surgery and stoma edema and inflammation. Ng et al. [44] showed no difference in the anastomotic leak or hospitalization between early and late stoma reversal, but early reversal increased SSI while late reversal increased stoma complications and worsened quality of life, aligning with our results. Although some guidelines support early reversal [45], some researchers favor late reversal for patients with temporary ileostomy for rectal cancer [46]. The timing of stoma reversal requires further large-scale studies to explore early versus late indications, impact on quality of life, postoperative complications, and other aspects of stoma reversal.

BMI is generally recognized as a risk factor for SSI development. According to the Cochrane Handbook recommendations, subgrouping and reducing heterogeneity when the types of original study variables are inconsistent [47]. Therefore, in this study, BMI was divided into continuous type variables and subtype variables for combined analysis. While BMI ≥ 25 kg/m^2^ was not statistically significant in the present study, data related to its continuous type variables were statistically significant. BMI ≥ 25 kg/m^2^ is a risk factor for incisional hernia after stoma reversal, but it is not clear whether this influence has an impact on postoperative incisional infection [48]. Gurunathan et al. [49] found the risk of postoperative incisional infection was 20% versus 50% higher in patients with BMI ≥ 25 kg/m^2^ versus BMI ≥ 30 kg/m^2^, consistent with this study’s results. This results in reduced blood and oxygen supply and delayed tissue regeneration. These patients also have inefficient metabolic regulation and a high inflammatory cascade, predisposing to incisional infection [50]. Some relevant studies have shown that BMI does not fully reflect body fat distribution, and that subcutaneous fat thickness is a better predictor of postoperative SSI in abdominal surgeries than BMI [51]. Fujii et al. [52] found subcutaneous fat thickness was the most valuable predictor of postoperative SSI in colorectal surgeries. The present study included two papers on the association of subcutaneous fat thickness with SSI incidence, with pooled results showing no statistical significance and high heterogeneity (I^2^ = 87.4%), possibly related to different study types. Future studies could further explore this factor’s effect on SSI.

This study found that the history of incisional infection after the initial (phase I) procedure impacts the development of incisional infection after stoma reversal. It is likely due to residual postoperative colonization with pathogenic microorganisms and susceptibility to pathogens. The specific mechanism by which a history of incisional infection after phase I surgery influences incisional infection after stoma reversal remains unknown; further relevant studies are needed [14,24]. The risk factors identified in our study can help us construct effective intervention programs, identify high-risk patients early, link the perioperative period of primary surgery to the perioperative period of stoma reversal, and drive the timing of prevention forward. In addition, the studies included in this article did not report risk factors such as diabetes, and we will further update the review to refine the factors influencing SR-SSI.

### Limitations

Although our study cannot clarify the causal relationship between the influencing factors and infection, it may provide a basis for subsequent prospective large cohort studies. Most studies included in this review were retrospective with weak arguments; larger, multicenter, prospective studies are needed. Inconsistent definitions of influencing factors and variable types across studies led to heterogeneity. Some influencing factors appeared in only two papers, limiting reliability and generalizability. Additionally, the data for the incidence of our study were all taken from the literature included in this paper, and data from other studies were not included, which may have had an impact.

## Conclusions

In summary, subcutaneous drainage, purse string closure, stoma type, inflammatory bowel disease, operative time, interval from stoma creation, BMI, and surgical site infection after primary surgery are influencing factors for after stoma reversal. Healthcare providers and policymakers can reduce the incidence of SR-SSI by increasing awareness of these risk factors, controlling modifiable risk variables, and developing effective intervention programs.

### Trial and protocol registration

Our protocol is registered with PROSPERO(ID: CRD42023449934) https://www.crd.york.ac.uk/PROSPERO/display_record.php?RecordID=449934.

## Supporting information

S1 File Supporting information. (ZIP)

S2 File PRISMA checklist. (PDF)
