# Correction: Risk factors and incidence of surgical wound infection after stoma reversal: A systematic review and meta-analysis

**DOI:** 10.1371/journal.pone.0336704

**Published:** 2025-11-11

**Authors:** Siruo Li, Ziyi Zhou, Feixia Wang, Weizhen Li, Chaoxu Liu, Lu Li

There are errors in the author affiliations. The correct affiliations are as follows:

Siruo Li^1^, Ziyi Zhou^1^, Feixia Wang^1^, Weizhen Li^1^, Chaoxu Liu^2^, Lu Li^1^

1 Nursing department, The First Affiliated Hospital, Zhejiang University School of Medicine, Hangzhou City, Zhejiang Province, China, 2 Colorectal Surgery Department, The First Affiliated Hospital, Zhejiang University School of Medicine, Hangzhou City, Zhejiang Province, China
