# Supplementary material for: Risk factors and incidence of surgical wound infection after stoma reversal: A systematic review and meta-analysis
Source: PLoS One. 2025 Jul 16;20(7):e0328344. doi: 10.1371/journal.pone.0328344 (PMC12266411; doi:10.1371/journal.pone.0328344)
Supplement: S1 File — (ZIP) [file pone.0328344.s001.zip › Supporting information/S1-S6 Files.pdf]

**Table S1 Search strategy and results of PubMed (searched on 30 July 2024)**

| # | Searches | Results |
|---|---|---|
| 1 | "ileostomy"[MeSH Terms] OR "ileostomy"[All Fields] OR "surgical stomas"[MeSH Terms] OR "stoma"[Text Word] OR "colostomy"[Title/Abstract] OR "ostomy"[Title/Abstract] OR "Loop Ileostomy"[Title/Abstract] OR "Diverting Ileostomy"[Title/Abstract] | 30,874 |
| 2 | "closure"[Title/Abstract] OR "reversal"[Title/Abstract] | 216,733 |
| 3 | "surgical wound infection"[MeSH Terms] OR "surgical wound infection"[Text Word] OR "complications"[Title/Abstract] OR "morbidity"[Title/Abstract] OR "surgical site infection"[Title/Abstract] OR "postoperative wound infection"[Title/Abstract] OR "SSI"[Title/Abstract] | 1,484,595 |
| 4 | "risk factor*"[Title/Abstract] OR "influencing factor*"[Title/Abstract] OR "predictor"[Title/Abstract] | 1,111,090 |
| 5 | #1 AND #2 AND #3 AND #4 | 258 |

**Table S2 Search strategy and results of Web of Science (searched on 30 July 2024)**

| # | Searches | Results |
|---|---|---|
| 1 | TI = ('Risk Factor*' OR 'Influencing Factor*' OR predictor) | 685,246 |
| 2 | AB = ('Risk Factor*' OR 'Influencing Factor*' OR predictor) | 3,929,021 |
| 3 | #1 OR #2 | 4,230,596 |
| 4 | TI = (complications OR morbidity OR 'Surgical Wound Infection*' OR 'Surgical Site Infections*' OR 'Postoperative Wound Infection' OR SSI) | 310,892 |
| 5 | AB = (complications OR morbidity OR 'Surgical Wound Infection*' OR 'Surgical Site Infections*' OR 'Postoperative Wound Infection' OR SSI) | 2,079,843 |
| 6 | #4 OR #5 | 2,238,568 |
| 7 | ' TI = (closure OR reversal) | 218,472 |
| 8 | AB = (closure OR reversal) | 642,400 |
| 9 | #7 OR #8 | 722,001 |
| 10 | TI = (ileostomy OR colostomy OR ostomy OR 'loop ileostomy' OR 'diverting ileostomy' OR stoma) | 21,057 |
| 11 | AB = (ileostomy OR colostomy OR ostomy OR 'loop ileostomy' OR 'diverting ileostomy' OR stoma) | 58,935 |
| 12 | #10 OR #11 | 67,054 |
| 13 | #3 AND #6 AND #9 AND #12 | 466 |

**Table S3 Search strategy and results of Cochrane Library (searched on 30 July 2024)**

| # | Searches | Results |
|---|---|---|
| 1 | MeSH descriptor: [Ileostomy] explode all trees | 316 |
| 2 | MeSH descriptor: [Colostomy] explode all trees | 265 |
| 3 | MeSH descriptor: [Ostomy] explode all trees | 2,236 |
| 4 | loop ileostomy OR diverting ileostomy OR stoma | 1,746 |
| 5 | #1 OR #2 OR #3 OR #4 | 3,642 |
| 6 | reversal | 5,741 |
| 7 | closure | 17,109 |
| 8 | #6 OR #7 | 22,593 |
| 9 | MeSH descriptor: [Postoperative Complications] explode all trees | 56,723 |
| 10 | MeSH descriptor: [Morbidity] explode all trees | 22,341 |
| 11 | MeSH descriptor: [Surgical Wound Infection] explode all trees | 4,665 |
| 12 | Postoperative Wound NEXT Infection* OR Surgical Site NEXT Infection* OR SSI | 9,797 |
| 13 | #9 OR #10 OR #11 OR #12 | 81,674 |
| 14 | MeSH descriptor: [Risk Factors] explode all trees | 38,726 |
| 15 | risk NEXT factor* OR Influencing NEXT factor* OR predictor | 112,225 |
| 16 | #14 OR #15 | 112,228 |
| 17 | #5 AND #8 AND #13 AND #16 | 34 |

**Table S4 Search strategy and results of Embase (searched on 30 July 2024)**

| # | Searches | Results |
|---|---|---|
| 1 | 'ileostomy' OR 'ileostomy'/exp OR ileostomy OR 'colostomy' OR 'colostomy'/exp OR colostomy OR 'ostomy' OR 'ostomy'/exp OR ostomy OR 'loop ileostomy'/exp OR 'loop ileostomy' OR 'diverting ileostomy'/exp OR 'diverting ileostomy' OR 'stoma' OR 'stoma'/exp OR stoma | 148,996 |
| 2 | closure OR reversal | 314,356 |
| 3 | 'complications'/exp OR complications OR 'morbidity'/exp OR morbidity OR 'surgical wound infection*' OR 'surgical site infections' OR 'postoperative wound infection*' OR ssi | 4,168,945 |
| 4 | 'risk factor*' OR 'influencing factor*' OR predictor | 2,309,876 |
| 5 | #1 AND #2 AND #3 AND #4 | 647 |

**Table S5 Search strategy and results of OpenGrey (searched on 30 July 2024)**

| # | Searches | Results |
|---|---|---|
| 1 | ("ileostomy" OR “colostomy” OR "Loop Ileostomy" OR "Diverting Ileostomy" OR "surgical stomas" OR stoma) AND (“closure” OR “reversal”) AND (“complications” OR “morbidity” OR "surgical wound infection" OR “Surgical Site Infection” OR “Postoperative Wound Infection” OR SSI) AND ("Risk Factor*" OR "Influencing Factor*" OR “predictor”) | 172 |

**Table S6 Exclusion reasons for full-text assessment**

| ID | Reference | Exclusion reasons |
|---|---|---|
| 1 | Risk factors in colostomy closure,1981 | No SSI was reported |
| 2 | Morbidity of closure of colostomy,1982 | Unable to get OR |
| 3 | Factors influencing the morbidity of colostomy closure,1982 | Unable to get OR |
| 4 | Complications of colostomy closure,1985 | Unable to get OR |
| 5 | Colostomy closure: still a hazardous procedure,1987 | Unable to get OR |
| 6 | Factors influencing the morbidity of colostomy closure,1988 | No SSI was reported |
| 7 | Protective colostomy closure: the hazards of a "minor" operation,1990 | No SSI was reported |
| 8 | Rates of morbidity and mortality after closure of loop and end colostomy,1990 | No SSI was reported |
| 9 | Colostomy closure: is it an intervention without risk? 1994 | No SSI was reported |
| 10 | Factors influencing the safety of colostomy closure in the elderly,1994 | Unable to get OR |
| 11 | Stoma closure and wound infection: an evaluation of risk factors,1995 | Wrong study population |
| 12 | The high morbidity of colostomy closure after trauma: Further support for the primary repair of colon injuries,1998 | Unable to get OR |
| 13 | Intestinal reconstruction after a Hartmann intervention: a high-risk procedure? 1998 | Unable to get OR |
| 14 | Colostomy closure: impact of preoperative risk factors on morbidity,1999 | No SSI was reported |
| 15 | Mortality and complications after stoma closure,2005 | Unable to get OR |
| 16 | Predictors for complications after loop stoma closure in patients with rectal cancer,2006 | No SSI was reported |
| 17 | Morbi-mortality of Hartmann's reversal procedure,2006 | No SSI was reported |
| 18 | Analysis of the factors related to the decision of restoring intestinal continuity after Hartmann's procedure,2007 | No SSI was reported |
| 19 | Reversal of Hartmann's procedure: a high-risk operation? 2007 | No SSI was reported |
| 20 | Morbidity and mortality after closure of loop ileostomy,2009 | No SSI was reported |
| 21 | Restoration of intestinal continuity after Hartmann's procedure--not a benign operation. Are there predictors for morbidity? 2011 | Wrong study population |
| 22 | Outcome of colostomy closure and influencing factors in patients with anorectal malformation ,2011 | No SSI was reported |
| 23 | Morbidity and mortality after the closure of a protective loop ileostomy: analysis of possible predictors,2012 | No SSI was reported |
| 24 | Relatively high incidence of complications after loop ileostomy reversal ,2012 | No SSI was reported |
| 25 | Hartmann reversal: obesity adversely impacts outcome,2012 | Unable to get OR |

| | | |
|---|---|---|
| 26 | Clinicopathologic Investigation of Surgical Site Infection in Stoma Closure,2012 | Wrong research type |
| 27 | Outcomes and predictors of surgical site infection (SSI) in stoma reversal,2012 | Duplicate data |
| 28 | Factors influencing postoperative adverse events after Hartmann's reversal,2012 | No SSI was reported |
| 29 | Morbidity related to defunctioning ileostomy closure after ileal pouch-anal anastomosis and low colonic anastomosis,2012 | Wrong study population |
| 30 | Morbidity associated with colostomy reversal after cytoreductive surgery and HIPEC,2014 | Wrong study population |
| 31 | The use of purse-string skin closure in loop ileostomy reversals leads to lower wound infection rates--a single high-volume centre experience,2014 | Unable to get OR |
| 32 | Predictors of Surgical Site Infection After Ostomy Reversal: Lessons From ACS-ASQIP,2014 | Duplicate data |
| 33 | Complications after Loop Ileostomy Closure: A Retrospective Analysis of 132 Patients,2014 | No SSI was reported |
| 34 | Body Mass Index as a Predictor of Postoperative Complications in Loop Ileostomy Closure after Rectal Resection in Japanese Patients,2014 | No SSI was reported |
| 35 | Morbidity after reversal of Hartmann operation: retrospective analysis of 56 patients,2015 | No SSI was reported |
| 36 | Morbidities after closure of ileostomy: analysis of risk factors, 2016 | No SSI was reported |
| 37 | Ileostomy closure by colorectal surgeons results in less major morbidity: results from an institutional change in practice and awareness,2016 | No SSI was reported |
| 38 | A Retrospective, Single-institution Review of Loop Ileostomy Reversal Outcomes ,2016 | Unable to get OR |
| 39 | Colostomy closure: risk factors for complications,2017 | No SSI was reported |
| 40 | High stoma prevalence and stoma reversal complications following anterior resection for rectal cancer: a population-based multicentre study,2017 | Wrong study population |
| 41 | Ileostomy reversal with handsewn techniques. Short-term outcomes in a teaching hospital,2017 | No SSI was reported |
| 42 | Risk factors for complications after diverting ileostomy closure in patients who have undergone rectal cancer surgery,2017 | No SSI was reported |
| 43 | Early closure of defunctioning stoma increases complications related to stoma closure after concurrent chemoradiotherapy and low anterior resection in patients with rectal cancer ,2017 | No SSI was reported |
| 44 | Postoperative Outcomes of Stoma Takedown: Results of Long-term Follow-up ,2017 | Unable to get OR |
| 45 | Evaluation of risk factors for complications after colostomy closure ,2019 | Unable to get OR |
| 46 | Colostomy reversal after a Hartmann's procedure Effects of experience on mortality and morbidity ,2019 | Unable to get OR |
| 47 | Factors affecting the morbidity and mortality of diverting stoma closure: retrospective cohort analysis of twelve-year period ,2019 | Unable to get OR |
| 48 | Prospective study on the safety and feasibility of early ileostomy closure 2 weeks after lower anterior resection for rectal cancer ,2019 | Wrong study population |
| 49 | Morbidity associated with closure of ileostomy after a three-stage ileal pouch-anal anastomosis ,2019 | Wrong study population |
| 50 | Investigating Risk Factors for Complications after Ileostomy Reversal in Low Anterior Rectal Resection Patients: An Observational Study ,2019 | No SSI was reported |
| 51 | Low level of albumin and longer interval to closure time increase the morbidities in ileostomy closure; A cohort study of 354 consecutive patients ,2020 | Duplicate data |
| 52 | Hartmann's reversal as a safe procedure for selected patients: analysis of 199 patients at a high-volume center in Sao Paulo ,2020 | Wrong study population |

| | | |
|---|---|---|
| 53 | Protective Loop Ileostomy Closure Techniques: Comparison of Three Different Surgical Techniques ,2020 | Wrong research type |
| 54 | Identification of risk factors for morbidity and mortality after Hartmann's reversal surgery - a retrospective study from two French centers ,2020 | No SSI was reported |
| 55 | Hartmann's reversal: factors affecting complications and outcomes,2020 | Wrong study population |
| 56 | Predictors of morbidity related to stoma closure after colorectal cancer surgery,2021 | No SSI was reported |
| 57 | Risk Factors for the Morbidity and Mortality of Stoma Closure,2021 | Unable to get OR |
| 58 | Association of Risk Factors for the Mortality and Morbidity of Stoma Closure,2021 | Unable to get OR |
| 59 | Outcomes of colostomy takedown following Hartmann's procedure: successful restoration of continuity comes with a high risk of morbidity ,2021 | Wrong study population |
| 60 | Does the timing of protective ileostomy closure post-low anterior resection have an impact on the outcome? A retrospective study,2021 | Unable to get OR |
| 61 | Closure of defunctioning loop ileostomy is associated with considerable morbidity,2012 | Duplicate data |
| 62 | Association between operative approach and complications in patients undergoing Hartmann's reversal,2013 | Duplicate data |
| 63 | Outcomes of diverting loop ileostomy reversal in the elderly: a case-control study,2021 | Unable to get OR |
| 64 | Prognostic factors for complications after loop ileostomy reversal,2022 | Unable to get OR |
| 65 | Comparison of surgical outcomes for colostomy closure performed by acute care surgeons versus a dedicated colorectal surgery service,2022 | Wrong study population |
| 66 | Open versus laparoscopic approach for Hartmann's reversal: Results of a 10-year retrospective cohort study,2022 | Wrong research type |
| 67 | A Study of Complications after Ileostomy Reversal in a Tertiary Care Center,2022 | Duplicate data |
| 68 | Incidence and risk factors associated with surgical site infection among patients that underwent stoma reversal at a Saudi tertiary hospital,2022 | Unable to get OR |
| 69 | Impact of chemotherapy on surgical outcomes in ileostomy reversal: a propensity score matching study from a single centre,2023 | Wrong research type |
| 70 | Complications following construction and closure of loop ileostomies,2011 | Wrong research type |
| 71 | Hartmann's reversal is associated with significantly higher morbidity compared to ileostomy reversal,2011 | Wrong research type |
| 72 | Postoperative complications and risk factors following diverting loop ileostomy closure,2022 | Wrong research type |
| 73 | Trial to reduce wound infection with contralateral drainage in loop ileostomy closure,2015 | Wrong research type |
| 74 | Does bowel stimulation before loop ileostomy closure reduce postoperative ileus? 2015 | Wrong research type |
| 75 | The wound infection after stoma closure between different methods of drainage,2019 | Wrong research type |
| 76 | Randomised controlled trial to assess efficacy of pelvic floor muscle training on bowel symptoms after low anterior resection for rectal cancer: study protocol,2021 | Wrong research type |
| 77 | Protocol for the UK cohort study to investigate the prevention of parastomal hernia (the CIPHER study),2021 | Wrong research type |
| 78 | The INTESTINE study: INtended TEmporary STomas In crohN's diseasE. Protocol for an international multicentre study,2022 | Wrong research type |
| 79 | Efferent Loop Stimulation Previous to Ileostomy Closure. Ileostim Trial,2022 | Wrong research type |
| 80 | Does investigation of an elevated postoperative C-reactive protein (CRP) detect infectious complications earlier following major colorectal surgery?2022 | Wrong research type |

**Table S7 Funding sources for 20 included studies**

| ID | Title | Funding sources |
|---|---|---|
| 1 | Complications of loop ileostomy closure in patients with rectal tumor | None |
| 2 | A beneficial effect of purse-string skin closure after ileostomy takedown: A retrospective cohort study | None |
| 3 | Factors predicting stomal wound closure infection rates | None |
| 4 | Association between incisional surgical site infection and the type of skin closure after stoma closure | None |
| 5 | Increased risk of complications in smokers undergoing reversal of diverting ileostomy | None |
| 6 | Risk factors for surgical site infection after stoma closure comparison between pursestring wound closure and conventional linear wound closure: Propensity score matching analysis | None |
| 7 | Risk Factors for Postoperative Complications Following Diverting Loop Ileostomy Takedown | None |
| 8 | Risk factor for the development of surgical site infection following ileostomy reversal: a single-center report | None |
| 9 | Prevalence of Surgical Site Infection at the Stoma Site following Four Skin Closure Techniques: A Retrospective Cohort Study | None |
| 10 | Predictors of complications from stoma closure in elective colorectal surgery: an assessment from the American College of Surgeons National Surgical Quality Improvement Program (ACSNSQIP) | None |
| 11 | Outcomes and predictors of incisional surgical site infection in stoma reversal | None |
| 12 | New Scoring System for Predicting the Risk of Surgical Site Infections Following Stoma Reversal | None |
| 13 | Multivariate analysis of risk factors for complications after loop ileostomy closure | None |
| 14 | Surgical site infections (SSIs) after stoma reversal (SR): risk factors, implications, and protective strategies | None |
| 15 | Subcutaneous vacuum drains reduce surgical site infection after primary closure of defunctioning ileostomy | None |
| 16 | Single-centre experience of loop ileostomy closure: a retrospective comparison of conventional-linear closure and purse-string closure on surgical-site-infection rates | None |
| 17 | Short-term outcome of diverting loop ileostomy reversals performed by residents: a retrospective cohort prognostic factor study | None |
| 18 | Risk factors for surgical site infections and trends in skin closure technique after diverting loop ileostomy reversal: A multi-institutional analysis | None//// |
| 19 | Loop Ileostomy Closure After Restorative Proctocolectomy: Outcome in 1,504 Patients | None |
| 20 | Clinical Benefits of Reducing Dead Space Using a Closed Suction Drain and Subcutaneous Large-bite Buried Suture Technique to Prevent Superficial Surgical-site Infections Following Primary Closure of a Diverting Stoma | None |

**Table S8** Quality assessment of various pooled analyses of surgical site infections after stoma reversal using the GRADE framework

| **Risk factors** | **Studies, *n*** | **Study design** | **Risk of bias** | **Inconsistency** | **Indirectness** | **Imprecision** | **Other considerations** | **Certainty** |
|---|---|---|---|---|---|---|---|---|
| Subcutaneous drainage | 3 | Observational | Not Serious | Serious[a] | Not Serious | Not Serious | Large magnitude of effect | Low |
| Suture method* | 6 | Observational | Not Serious | Not Serious | Not Serious | Not Serious | None | Low |
| Incision type | 2 | Observational | Not Serious | Serious[a] | Not Serious | Not Serious | None | Very low |
| Subcutaneous fat thickness | 2 | Observational | Not Serious | Serious[a] | Not Serious | Serious[b] | None | Very Low |
| History of fascia cracking | 2 | Observational | Not Serious | Serious[a] | Not Serious | Serious[b] | None | Very low |

| | | | | | | | | |
|---|---|---|---|---|---|---|---|---|
| Stoma type* | 5 | Observational | Not Serious | Serious[a] | Not Serious | Not Serious | None | Very low |
| Inflammatory bowel disease | 3 | Observational | Not Serious | Not serious | Not Serious | Not Serious | None | Low |
| Cancer | 2 | Observational | Not Serious | Not serious | Not Serious | Not Serious | None | Low |
| Diverticulosis | 2 | Observational | Not Serious | Serious[a] | Not Serious | Serious[b] | None | Very Low |
| Operation time（> 60min） | 3 | Observational | Not Serious | Not serious | Not Serious | Not Serious | None | Low |
| Operation time（continued） | 2 | Observational | Not Serious | Not Serious | Not Serious | Not Serious | None | Very Low |
| Period from stoma creation* | 2 | Observational | Not Serious | Not serious | Not Serious | Not serious | Large magnitude of effect | Low |
| Period from stoma creation（continued） | 2 | Observational | Not Serious | Serious[a] | Not Serious | Not serious | None | Very Low |

| | | | | | | | | |
|---|---|---|---|---|---|---|---|---|
| Smoke | 5 | Observational | Not Serious | Serious[a] | Not Serious | Not Serious | None | Very low |
| BMI（≥25） | 3 | Observational | Not Serious | Serious[a] | Not Serious | Not Serious | None | Very low |
| BMI (continued） | 2 | Observational | Not Serious | Not Serious | Not Serious | Not Serious | None | Low |
| Gender* | 4 | Observational | Not Serious | Serious[a] | Not Serious | Not Serious | None | Very Low |
| Surgical site infection after primary surgery | 2 | Observational | Not Serious | Not Serious | Not Serious | Not Serious | None | Low |

*Note* GRADE=Grading of Recommendations, Assessment, Development, and Evaluation

a. $I^2$ value >50

b. Wide confidence intervals

# PRISMA 2020 Checklist

| Section and Topic | Item # | Checklist item | Location where item is reported |
|---|---|---|---|
| **TITLE** | | | |
| Title | 1 | Identify the report as a systematic review. | 1 |
| **ABSTRACT** | | | |
| Abstract | 2 | See the PRISMA 2020 for Abstracts checklist. | 2 |
| **INTRODUCTION** | | | |
| Rationale | 3 | Describe the rationale for the review in the context of existing knowledge. | 2-3 |
| Objectives | 4 | Provide an explicit statement of the objective(s) or question(s) the review addresses. | 2-3 |
| **METHODS** | | | |
| Eligibility criteria | 5 | Specify the inclusion and exclusion criteria for the review and how studies were grouped for the syntheses. | 4 |
| Information sources | 6 | Specify all databases, registers, websites, organisations, reference lists and other sources searched or consulted to identify studies. Specify the date when each source was last searched or consulted. | 3-4 |
| Search strategy | 7 | Present the full search strategies for all databases, registers and websites, including any filters and limits used. | 28-29 |
| Selection process | 8 | Specify the methods used to decide whether a study met the inclusion criteria of the review, including how many reviewers screened each record and each report retrieved, whether they worked independently, and if applicable, details of automation tools used in the process. | 5 |
| Data collection process | 9 | Specify the methods used to collect data from reports, including how many reviewers collected data from each report, whether they worked independently, any processes for obtaining or confirming data from study investigators, and if applicable, details of automation tools used in the process. | 5 |
| Data items | 10a | List and define all outcomes for which data were sought. Specify whether all results that were compatible with each outcome domain in each study were sought (e.g. for all measures, time points, analyses), and if not, the methods used to decide which results to collect. | 4-5 |
| | 10b | List and define all other variables for which data were sought (e.g. participant and intervention characteristics, funding sources). Describe any assumptions made about any missing or unclear information. | 4-5 |
| Study risk of bias assessment | 11 | Specify the methods used to assess risk of bias in the included studies, including details of the tool(s) used, how many reviewers assessed each study and whether they worked independently, and if applicable, details of automation tools used in the process. | 5 |
| Effect measures | 12 | Specify for each outcome the effect measure(s) (e.g. risk ratio, mean difference) used in the synthesis or presentation of results. | 6 |
| Synthesis methods | 13a | Describe the processes used to decide which studies were eligible for each synthesis (e.g. tabulating the study intervention characteristics and comparing against the planned groups for each synthesis (item #5)). | 6 |
| | 13b | Describe any methods required to prepare the data for presentation or synthesis, such as handling of missing summary statistics, or data conversions. | 6 |
| | 13c | Describe any methods used to tabulate or visually display results of individual studies and syntheses. | 6 |
| | 13d | Describe any methods used to synthesize results and provide a rationale for the choice(s). If meta-analysis was performed, describe the model(s), method(s) to identify the presence and extent of statistical heterogeneity, and software package(s) used. | 6 |
| | 13e | Describe any methods used to explore possible causes of heterogeneity among study results (e.g. subgroup analysis, meta-regression). | 6 |
| | 13f | Describe any sensitivity analyses conducted to assess robustness of the synthesized results. | 6 |
| Reporting bias assessment | 14 | Describe any methods used to assess risk of bias due to missing results in a synthesis (arising from reporting biases). | 6 |
| Certainty assessment | 15 | Describe any methods used to assess certainty (or confidence) in the body of evidence for an outcome. | 5 |

# PRISMA 2020 Checklist

| Section and Topic | Item # | Checklist item | Location where item is reported |
|---|---|---|---|
| **RESULTS** | | | |
| Study selection | 16a | Describe the results of the search and selection process, from the number of records identified in the search to the number of studies included in the review, ideally using a flow diagram. | **7** |
| | 16b | Cite studies that might appear to meet the inclusion criteria, but which were excluded, and explain why they were excluded. | **28-29** |
| Study characteristics | 17 | Cite each included study and present its characteristics. | **7-10** |
| Risk of bias in studies | 18 | Present assessments of risk of bias for each included study. | **7-10** |
| Results of individual studies | 19 | For all outcomes, present, for each study: (a) summary statistics for each group (where appropriate) and (b) an effect estimate and its precision (e.g. confidence/credible interval), ideally using structured tables or plots. | **11-18** |
| Results of syntheses | 20a | For each synthesis, briefly summarise the characteristics and risk of bias among contributing studies. | **11-18** |
| | 20b | Present results of all statistical syntheses conducted. If meta-analysis was done, present for each the summary estimate and its precision (e.g. confidence/credible interval) and measures of statistical heterogeneity. If comparing groups, describe the direction of the effect. | **8-10** |
| | 20c | Present results of all investigations of possible causes of heterogeneity among study results. | **11-18** |
| | 20d | Present results of all sensitivity analyses conducted to assess the robustness of the synthesized results. | **17-18** |
| Reporting biases | 21 | Present assessments of risk of bias due to missing results (arising from reporting biases) for each synthesis assessed. | **17** |
| Certainty of evidence | 22 | Present assessments of certainty (or confidence) in the body of evidence for each outcome assessed. | **17-18** |
| **DISCUSSION** | | | |
| Discussion | 23a | Provide a general interpretation of the results in the context of other evidence. | **19-23** |
| | 23b | Discuss any limitations of the evidence included in the review. | **23** |
| | 23c | Discuss any limitations of the review processes used. | **23** |
| | 23d | Discuss implications of the results for practice, policy, and future research. | **19-23** |
| **OTHER INFORMATION** | | | |
| Registration and protocol | 24a | Provide registration information for the review, including register name and registration number, or state that the review was not registered. | **3** |
| | 24b | Indicate where the review protocol can be accessed, or state that a protocol was not prepared. | **24** |
| | 24c | Describe and explain any amendments to information provided at registration or in the protocol. | **24** |
| Support | 25 | Describe sources of financial or non-financial support for the review, and the role of the funders or sponsors in the review. | **28-29** |
| Competing interests | 26 | Declare any competing interests of review authors. | **23** |
| Availability of data, code and other materials | 27 | Report which of the following are publicly available and where they can be found: template data collection forms; data extracted from included studies; data used for all analyses; analytic code; any other materials used in the review. | **23** |

*From:* Page MJ, McKenzie JE, Bossuyt PM, Boutron I, Hoffmann TC, Mulrow CD, et al. The PRISMA 2020 statement: an updated guideline for reporting systematic reviews. BMJ 2021;372:n71. doi: 10.1136/bmj.n71

For more information, visit: http://www.prisma-statement.org/
