# Supplementary figures and images for: Risk factors and incidence of surgical wound infection after stoma reversal: A systematic review and meta-analysis
Source: PLoS One. 2025 Jul 16;20(7):e0328344. doi: 10.1371/journal.pone.0328344 (PMC12266411; doi:10.1371/journal.pone.0328344)

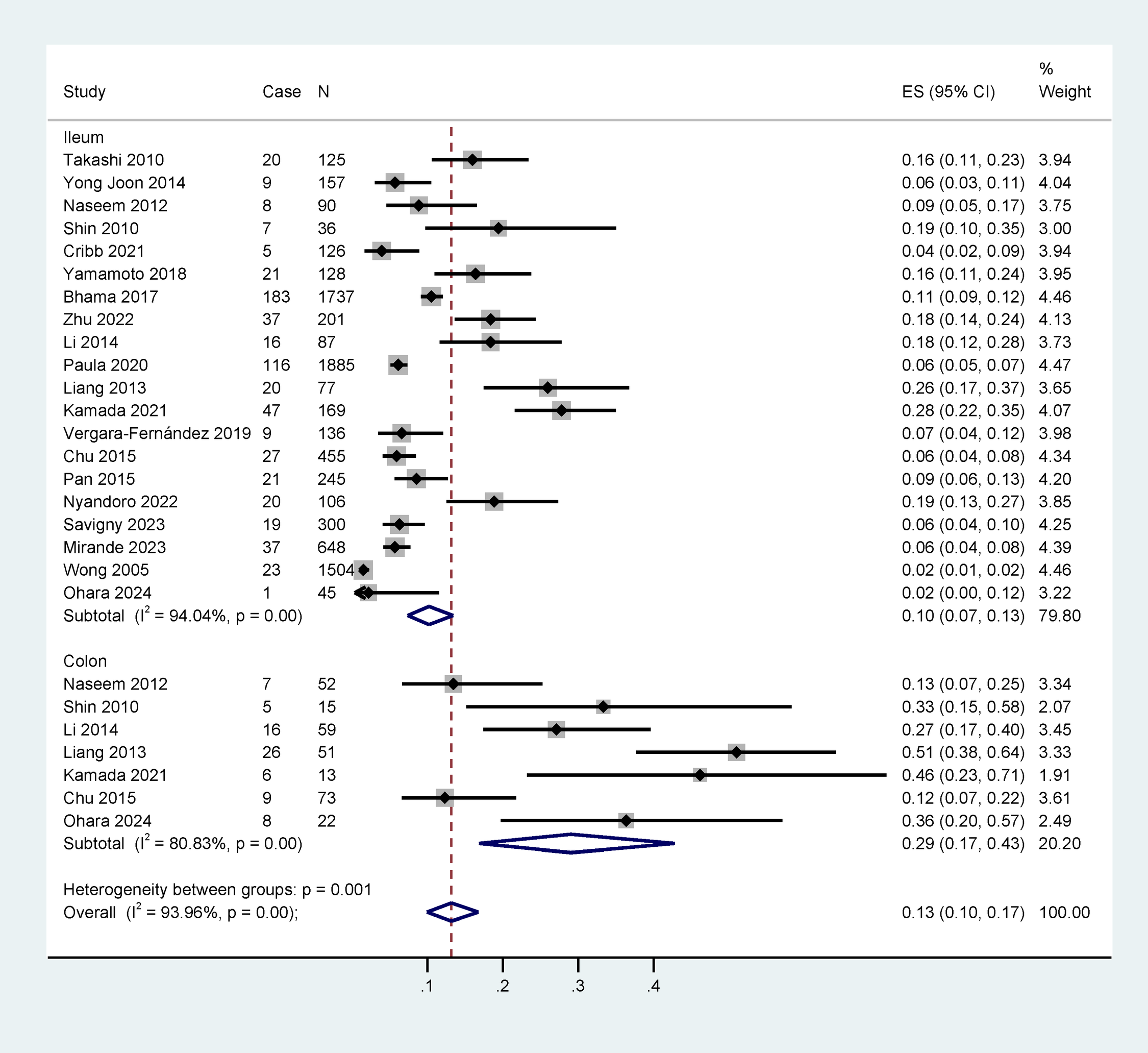

Supplement: S1 File — (ZIP) [file pone.0328344.s001.zip › Supporting information/S1_Fig.tif]

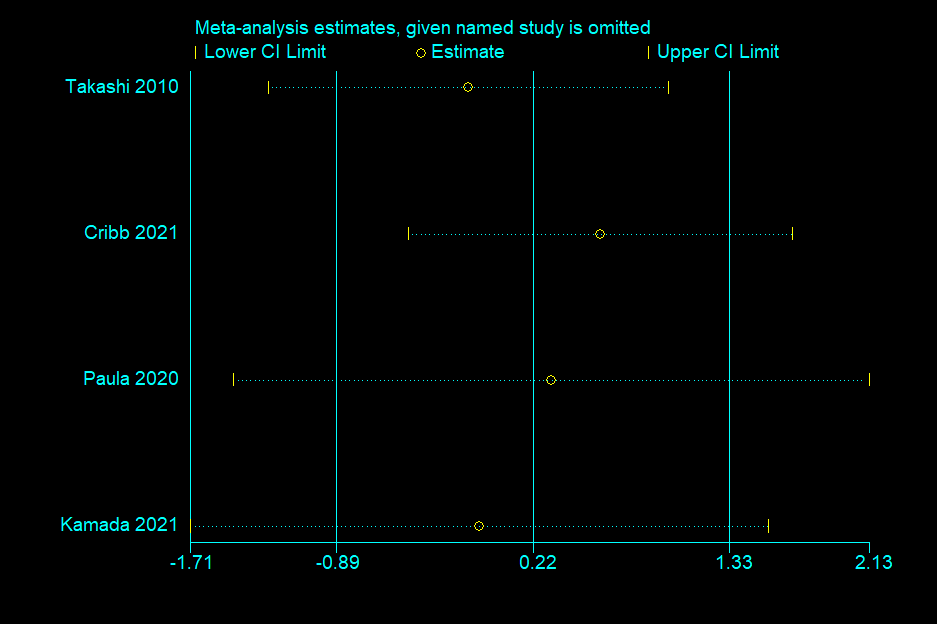

Supplement: S1 File — (ZIP) [file pone.0328344.s001.zip › Supporting information/S10_Fig.tif]

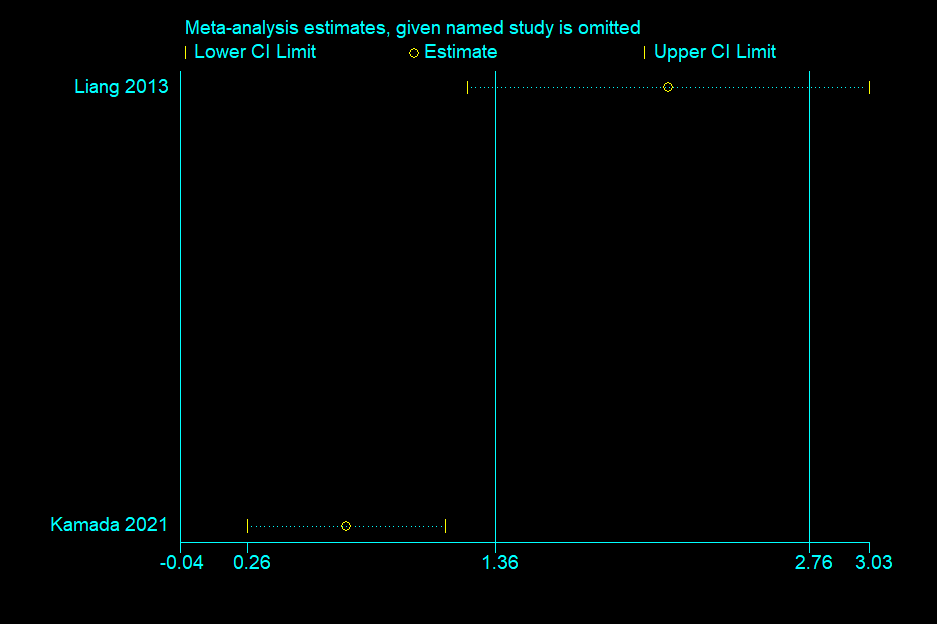

Supplement: S1 File — (ZIP) [file pone.0328344.s001.zip › Supporting information/S11_Fig.tif]

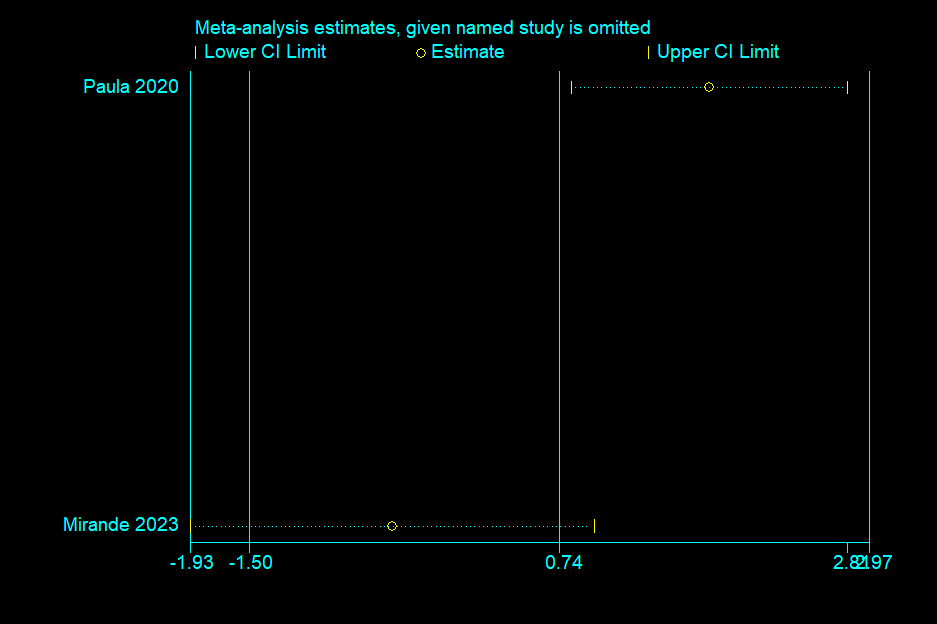

Supplement: S1 File — (ZIP) [file pone.0328344.s001.zip › Supporting information/S12_Fig.tif]

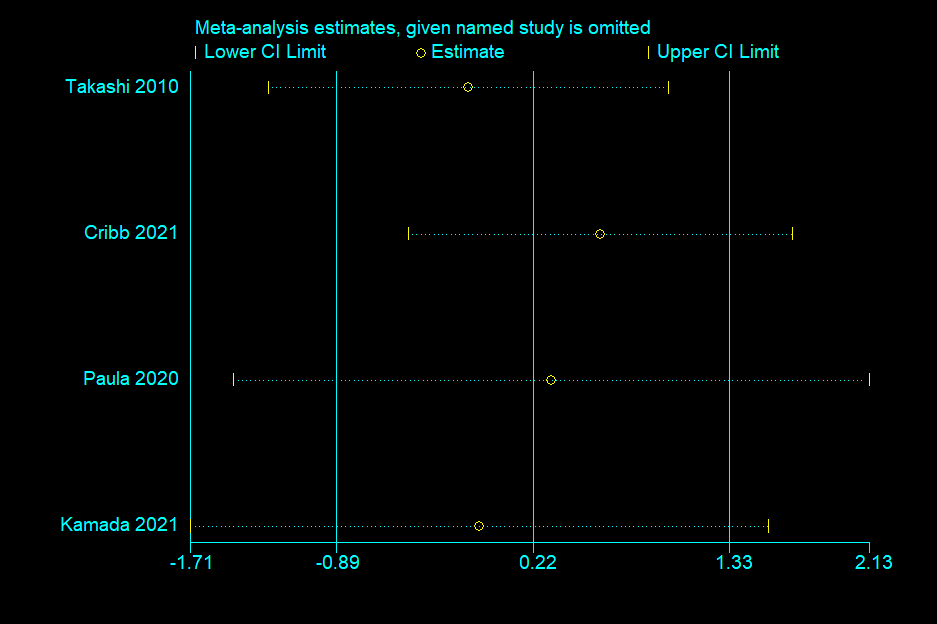

Supplement: S1 File — (ZIP) [file pone.0328344.s001.zip › Supporting information/S13_Fig.tif]

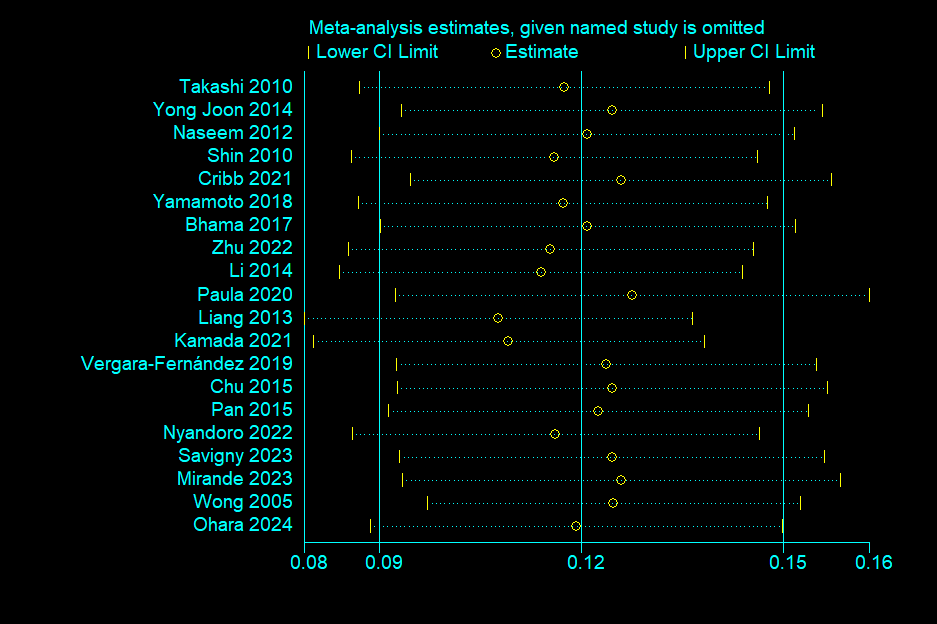

Supplement: S1 File — (ZIP) [file pone.0328344.s001.zip › Supporting information/S2_Fig.tif]

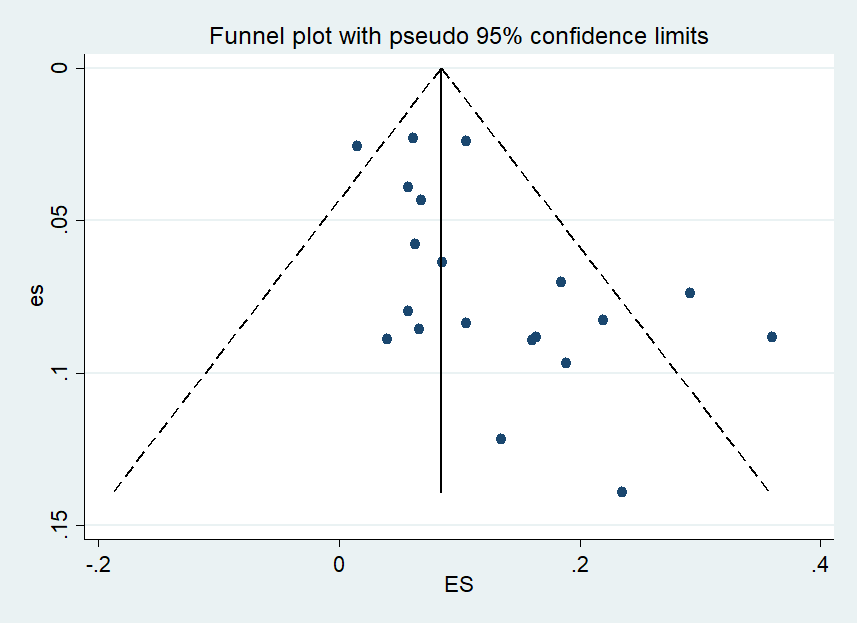

Supplement: S1 File — (ZIP) [file pone.0328344.s001.zip › Supporting information/S3_Fig.tif]

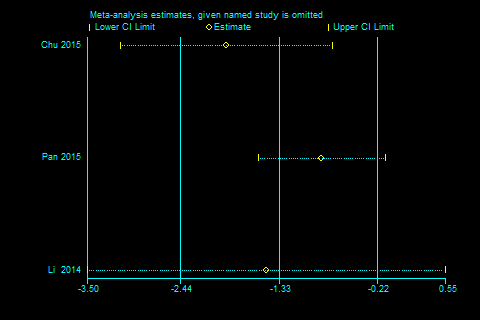

Supplement: S1 File — (ZIP) [file pone.0328344.s001.zip › Supporting information/S4_Fig.tif]

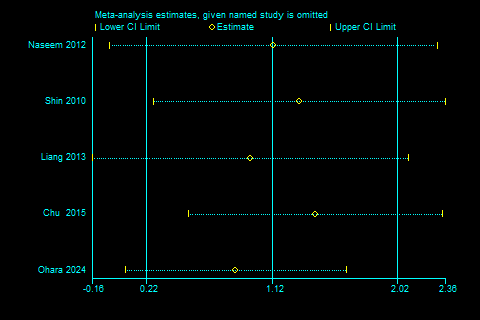

Supplement: S1 File — (ZIP) [file pone.0328344.s001.zip › Supporting information/S5_Fig.tif]

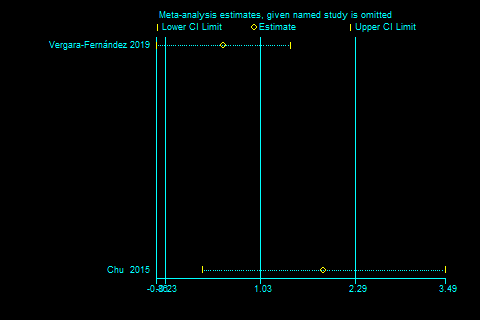

Supplement: S1 File — (ZIP) [file pone.0328344.s001.zip › Supporting information/S6_Fig.tif]

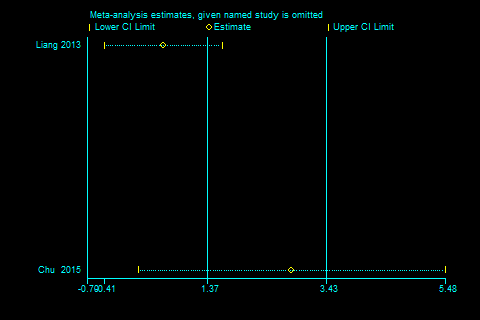

Supplement: S1 File — (ZIP) [file pone.0328344.s001.zip › Supporting information/S7_Fig.tif]

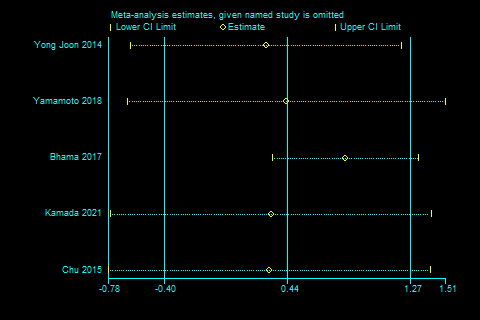

Supplement: S1 File — (ZIP) [file pone.0328344.s001.zip › Supporting information/S8_Fig.tif]

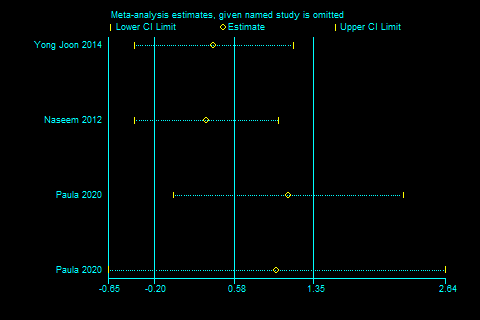

Supplement: S1 File — (ZIP) [file pone.0328344.s001.zip › Supporting information/S9_Fig.tif]
